# The XXXIX Long Fox Memorial Lecture

**Published:** 1951-01

**Authors:** William Savage


THE XXXIX LONG FOX MEMORIAL LECTURE

*Delivered in the University on October 17th, 1950*

BY

SIR WILLIAM SAVAGE, M.D., B.SC.

ON

### SOME FOOD INFECTION PROBLEMS

---

For each food infection outbreak we can postulate four essential factors. Two obvious ones are the organism responsible and the particular vehicle through which the organism gains access to man. Equally important is the reservoir from which the organism gained access to the food vehicle and the fourth factor is the path taken from the reservoir to the vehicle (food or water, which also is a food). No outbreak can be said to be fully explained unless all four factors have been ascertained. Time will not permit a complete discussion on all food infections, but I hope to deal with the two most important groups: the enteric group and acute food poisoning.

### THE ENTERIC GROUP

The three recognized types are: typhoid fever, due to the typhoid bacillus; paratyphoid fever due to B. paratyphosum; and dysentery infections, for which various strains of dysentery bacilli are responsible. I shall not be able to discuss dysentery infections, but many outbreaks have been spread by foods. Their severity closely depends upon the strain responsible. The comparatively rare food outbreaks due to the Flexner strain may be severe with many deaths while the much commoner Sonne outbreaks are mild and usually with little or no mortality, as in one I investigated spread by ice cream almost certainly infected in preparation.

*Typhoid fever* and paratyphoid fever are clinically much alike, but the latter is much milder and with a low fatality rate. They show important differences however in the ways they are spread. Until recent years typhoid fever was essentially regarded as a water-spread disease, with milk a poor second, with shell-fish looming as an important vehicle of spread, while foods other than milk rarely were involved. To-day I think we can record a change of emphasis. Water is a less predominant vehicle although, of course, still of primary importance, witness the extensive water-spread Croydon outbreak. So far as records go shell-fish (oysters, cockles, mussels) are rare vehicles: this is a tribute to our Public Health control over water and shell-fish.

*Paratyphoid.* It is of the greatest interest that in the allied disease paratyphoid we find very different vehicles. In a paper published in 1942 on an epidemiological study of 40 outbreaks of paratyphoid fever the vehicle in no less than 17 (42 per cent.) was cream cakes or other confectionery, 14 were associated with other foods (milk 8, ice cream 2, other foods 4), water only 2, direct case to case infection 3, not ascertained 4. Turning to records available to me in Great Britain since my report was published of 11 typhoid fever outbreaks, the vehicles were water 3, milk 3, confectionery 0, other foods 4, doubtful 1. The 9 paratyphoid outbreaks were water 0, milk 3, confectionery 3, infection from a contaminated bathing-pool 1, doubtful 2. The same sort of differences are maintained, but clearly typhoid fever is becoming more food spread. I have not noted dairy products as vehicles in either disease in this country but in Austria fairly recently, butter has caused three outbreaks and cheese a good many outbreaks in U.S.A. and Canada, all of them being typhoid fever.

These two diseases are particularly interesting to-day because recent bacteriological work has given us bacteriological weapons which enable their epidemiology to be studied with an intensity and accuracy quite impossible only a few years ago. One such aid is that the methods for the detection and isolation of both. typhoid and paratyphoid bacilli, not only from human sources (excreta, urine, etc.), but from suspected vehicles such as water, sewage effluents, foods and the like, are so enormously improved that what was once a hopeless task now yields a good percentage of positive results. A second advance is the ability we now possess to divide both organisms into a number of types and, using the proper procedure, to determine the type of the strain responsible for the outbreak. This is a valuable aid to linking up cases with vehicles and reservoirs, and has yielded brilliant results. A third weapon is that we now have much more reliable means of ascertaining if a person is a carrier of these organisms, not only by cultural tests, but by the presence of special antibodies in the blood (vi-antibody). The importance of this is referred to later on. We owe a great debt to Dr. Felix for his work on this aspect and on phage typing.

When we turn to the reservoirs of infection, we regularly meet a carrier of the responsible bacillus. In typhoid fever this is practically always a chronic carrier, but in paratyphoid fever often the carrier is not a chronic but a temporary one or may be an infected person with few or no symptoms. The distinction is of some importance and merits some explanation. In cases of either disease the bacilli tend to persist in the body and to be excreted in faeces, urine or both long after all clinical symptoms have subsided. There is, however, a gradual and usually an orderly reduction of this infection with time and most cases have cleared up at the end of a few months. In some 3 per cent. of both diseases the bacilli persist for years and these are the chronic carriers. In paratyphoid outbreaks it is common to have many persons who are infected, but who do not develop recognizable symptoms. These persons carry the bacilli, but fortunately for only limited periods, and form the transitory carrier group. So far as my studies go they are an important source of infection in paratyphoid fever and may be more important than the chronic carrier.

In many outbreaks the chain is complete, i.e. the reservoir, a chronic or temporary carrier, the path is the carrier infecting food or water, which then is the vehicle. The problem of prevention involves destroying one or more of these links in the chain. Obviously the main link to be snapped is the reservoir, for in practically every outbreak the reservoir is a carrier. Two lines suggest themselves for preventive measures.

One is to examine every person associated with work which facilitates the transmission of infection, such as all persons concerned with milk and workers handling food in preparation and in distribution. This procedure is not really practicable. To ascertain whether a person is or is not a carrier is a long and complicated business needing repeated bacteriological examinations of excreta, etc., and requiring also the willing co-operation of the workers. When we also realize the frequent changes of staff, this method as a routine procedure is quite impracticable: and examinations less complete are useless and give a false sense of security. We can, however, minimize the risk of a carrier being a source of infection by inculcating a high standard of personal hygiene, the most important item being invariable washing of hands after use of the closet. Facilities must be provided to make this an easy practice.

There is one line of prevention which is very hopeful. I explained above that only some 3 per cent. of typhoid or paratyphoid cases become chronic carriers and the other 97 per cent. are safe. Detect and control these 3 per cent. of chronic carriers and we go a long way to reducing risks from the carriers. At long last something is being done in this direction and this is possible because of one recent bacteriological advance. Experience shows that the absence of vi-antibodies late in the disease coincides with absence of the carrier state. It is a recommendation of the Ministry of Health since 1946, that before discharge from hospital every convalescent should have a blood test for vi-agglutinins. If they are absent no further action need be taken. If present, and in early stages of convalescence nearly half may be positive, then a second test should be made three months later: and if still positive a detailed bacteriological investigation made. In this way the persistent carriers would be weeded out. When found steps can be taken to prevent them working in businesses where infection can be spread and there are powers to this end under the Public Health (Infectious Diseases) Regulations, 1927. This is as yet not a compulsory procedure and is being tried out experimentally, but it is on sound lines. For technical reasons we know that by no means all chronic carriers of these two diseases will be detected: but if we only detect a material proportion it will be a most important preventive measure.

### ACUTE FOOD POISONING

The same four factors in causation operate, but the clinical picture is very different and vehicles, reservoirs and paths are unlike those found with the enteric infections. Excluding the rare botulism there are two main types of bacterial food poisoning which include the great majority of outbreaks in this country. The organisms responsible in one group are strains of salmonella bacteria and in the other a special type of staphylococcus which produces enterotoxin.

*Salmonella* food poisoning. When I started my investigations on this group, nearly fifty years ago, only three different types in the group were known: now they muster three hundred or more and the list is constantly added to. Apart from the one type (B. paratyphosum) they are essentially a group which affects the lower animals (including birds). They set up infections in many animals: cattle, rodents, pigs, horses and many birds including ducks and hens. Recently they have been isolated from cats and from dogs. Their main reservoir is an animal source and animals are found as carriers after an infection. Different Salmonella types seem to have a predilection for certain animal groups. To give one example, S. dublin is a type fairly widespread as a cause of disease in young cattle and may persist in the carrier state. A good many outbreaks of food poisoning are on record associated with this type and with milk as the vehicle, the path to the milk being the bacilli excreted into the milk or in infected dung contaminating the milk.

For many years I have taught that (apart from paratyphoid fever) chronic carriers of salmonella are not met with in man, since when infection occurs they act as intense irritants and this leads to their elimination within a period reckoned in weeks. As regards acute food poisoning I believe this is broadly true but, as explained below, tends to become less correct. An examination of salmonella food poisoning outbreaks shows the extreme rarity of human chronic carriers. With our improved cultural methods, persons are more frequently being found harbouring salmonella strains, but all or most seem to be recent infections and not the cause of the outbreak. My own opinion is that the salmonella group is in a stage of evolution. It may well be that, as in animals, more types are becoming invasive to man, with the possibility of some enduring as chronic carriers: but much more evidence is needed.

In these salmonella food poisoning outbreaks an animal source is the most probable reservoir, but it is an established fact that after a human attack total elimination takes some weeks and such temporary carriers may act as infective agencies. The path to the vehicle infected includes actually infected ducks' eggs, contamination of food from excreta of rats and mice or of other infected animals (dogs, cats), from infected domestic animals such as cattle or pigs and dried eggs contaminated with salmonella strains. Any food can serve as the vehicle, but the highest incidence is upon handled and manipulated foods like brawn, meat pies, sausages, pressed meat, ice cream and also milk. Naturally, preventive measures must include steps to control salmonella animal infections and to prevent contamination of food from the sources indicated. Special precautions are necessary in the preparation of these made-up foods, and particularly to insist on rapid cooling of all heated foods which may carry infection, to prevent multiplication of salmonella strains in the warm food.

*Staphylococcus enterotoxin.* These very characteristic outbreaks are due to preformed toxin and not to any infection of the human subject. The poison being all formed, the symptoms come on very rapidly after the food is consumed (two–four hours frequently) and are acute and severe; nausea, vomiting, diarrhoea, severe abdominal pain and collapse being the main signs. The sufferer may feel he is going to die, but practically never does and recovery is rapid.

Our great problem in prevention is that staphylococci are everywhere and we all harbour millions of them, but it is only a very special type which produces this virulent toxin, the so-called enterotoxin, which is a potential culprit. Tests on monkeys have some validity as a differential test and some workers consider a kitten test serves to distinguish this strain, but in this country we have not found it reliable. The only test we can really rely upon is to grow the suspected staphylococcus in a suitable liquid medium, sterilize by filtration and then feed to a human volunteer. If he gets an attack of food poisoning it is the real thing, but so are the effects on the human victim. Obviously until we are able to study the origins and distribution of this type our preventive efforts are greatly hampered. A simple and reliable differentiation test would be of the utmost service.

The reservoirs of infection are essentially human sources, such as the unhealthy nose and throat and possibly also the healthy nose and throat, discharging ears, sores on the skin in places where they come in contact with food and indeed any local infections. Not infrequently such a toxic strain is found under the finger nails. The paths from these reservoirs to food are obvious and impress the need for measures to exclude persons suffering from the above-mentioned abnormal conditions from handling food for human consumption.

The vehicles vary widely, but prominent are cakes (cream and custard filled) and processed and made-up meat. The second group have been prominent of recent years in this country. A very important fact is that it takes a considerable dose of this toxin to cause symptoms. Again and again we find a food (trifle, cakes, etc.) made overnight and kept in a warm kitchen, harmless when some is eaten soon after preparation, but served next day at a function responsible for an extensive and severe outbreak. There is the same need for rapid cooling after preparation and storage at refrigerator temperature until usage as mentioned for Salmonella infections.

In conclusion, the lecturer emphasized that we have the requisite knowledge: prevention lags not from ignorance but mainly because of administrative difficulties.

